# Circular validation in diagnostic research: re-examination of a widely cited study on primary hemostasis screening

**DOI:** 10.3389/fmed.2026.1909484

**Published:** 2026-10-29

**Authors:** Andreas Calatzis, Michael Spannagl, Georg Wittmann

**Affiliations:** 1 Institute for Cardiovascular Prevention (IPEK), Ludwig-Maximilians-Universität München, Munich, Germany; 2 Institute for Laboratory Medicine, LMU University Hospital, Munich, Germany; 3 Labor Becker MVZ, Munich, Germany

**Keywords:** incorporation bias, PFA-100, PFA-200, platelet function testing, verification bias

## Abstract

**Introduction:**

A 2004 study reported high sensitivities for laboratory assays used in the preoperative detection of primary hemostasis disorders and has since been widely cited. A re-evaluation of its methodological validity is performed.

**Methods:**

The study design and calculations were reviewed focusing on the definition of “impaired hemostasis” and the derivation of diagnostic performance. A synthetic dataset of random values was generated, and assay-specific cut-offs were defined to reproduce the proportions of positive results reported in the original study. The detection of “impaired hemostasis” was then calculated using a self-referential approach.

**Results:**

Three sets of performance figures are provided in the 2004 study. In one set, the results of the evaluated tests were used to define the reference standard against which their own sensitivities and specificities were then calculated (incorporation bias). For one assay a second set of performance figures was reported. The index test had been performed first in all patients; the further diagnostic work-up and the classification of clinical findings depended on its result (verification bias). The cut-off used for the index test was determined *post hoc* by ROC analysis, which often inflates performance metrics. The sensitivity and specificity values reported for the individual questionnaire items are likewise affected by incorporation bias. The synthetic dataset reproduced the reported detection rates despite consisting of random numbers.

**Conclusion:**

The widely cited results of the 2004 study cannot be used as evidence of diagnostic accuracy, because the reference standard was established using the very tests whose performance was then evaluated against it.

## Introduction

In a study published in 2004 (1), a prospective cohort of 5,649 patients was used to evaluate a strategy for preoperative screening of primary hemostasis disorders based on medical history and laboratory testing. A standardized questionnaire, in combination with clinical assessment, identified a positive bleeding history in 11.2% of patients (*n* = 628). Laboratory evaluation included platelet function testing (PFA collagen–epinephrine and collagen–ADP), bleeding time, von Willebrand factor antigen (vWF:Ag), activated partial thromboplastin time (aPTT), prothrombin time (PT), and platelet count. The study reported that the PFA collagen–epinephrine test identified 97.7% of patients with “primary hemostasis disorders”, PFA collagen–ADP identified 77.7% and vWF:Ag detected 15.2%. In addition, based on a receiver operating characteristic (ROC) analysis, a sensitivity of 90.8% and a specificity of 86.6% were reported for the PFA collagen–epinephrine test.

According to the “Web of Science” the study has been cited 150 times (2). It has been cited by articles published in the British Journal of Anaesthesia (3), Anesthesiology (4), the American Journal of Hematology (5), the British Journal of Haematology (6), and the European Journal of Anaesthesiology (7). It has also been referenced in publications by authors of the present article (8), multidisciplinary recommendations (9), clinical practice guidelines (7, 10), and educational articles on patient management (11, 12). Furthermore, the study was included in the instructions for use of the PFA collagen–ADP and collagen–epinephrine tests (13, 14). In several studies (15, 16), discrepancies between the findings of the 2004 study and newer data have been critically appraised.

Thus, the 2004 publication may appear to represent a pivotal study with lasting influence and important conclusions. However, as will be demonstrated, its estimates of sensitivity and specificity for laboratory tests in primary hemostasis disorders are affected by what is known in diagnostic accuracy research as incorporation bias (17, 18): the results of the assays under evaluation were used to define the presence of the target condition and then to derive their own diagnostic performance, so that no reference standard independent of the index tests existed. In plain terms, the validation is circular.

The aims of this Perspective are a) to communicate this finding, b) to analyze lessons and c) to derive proposals, to avoid such situations for the future.

## Analysis of the calculations used in the 2004 study

A total of 5,649 patients were included. Platelet count, aPTT, PT, and PFA (collagen–ADP and collagen–epinephrine) were performed in all patients. Based on medical history, clinical examination, and a standardized questionnaire, patients were classified as having either a positive or negative bleeding history (1).

In patients classified as having a positive bleeding history, additional testing — including bleeding time and von Willebrand factor antigen (vWF:Ag) — was performed. Overall, 628 patients (11.2%) were classified as having a positive bleeding history (Fig. 1 in the 2004 publication). Of these, 256 exhibited at least one abnormal test result and were therefore classified as having “impaired hemostasis” (Fig. 2 in the 2004 publication). Among patients classified as having “impaired hemostasis”, 250 showed an abnormal PFA collagen–epinephrine result, 199 an abnormal PFA collagen–ADP result, 188 a prolonged bleeding time, 39 an abnormal vWF:Ag, 24 an abnormal aPTT, 10 an abnormal PT, and 2 an abnormal platelet count.

So far, so good. If the study had been purely observational, describing coagulation abnormalities using previously validated assays, the design would have been acceptable.

However, in a subsequent step, sensitivities and specificities were calculated for the assays with respect to detecting “impaired hemostasis,” a condition that had been defined using the same test results. In the terminology of diagnostic accuracy research, this constitutes incorporation bias: the index test forms part of the reference standard against which its performance is evaluated (17, 18). As a result, agreement between the test and the reference standard is built into the design rather than measured. Estimates of sensitivity and specificity require a reference standard that is independent of the index test (19).

Similar problems affect Table 3 of the 2004 study, which reports sensitivity, specificity and predictive value for each of the 12 questions of the bleeding questionnaire with respect to “impaired hemostasis” as defined in the article. A positive bleeding history — defined by a positive answer to at least one question, or by clinical evidence — triggered additional testing that could establish “impaired hemostasis”, whereas patients who answered all questions negatively were not investigated further. Each questionnaire item was thus evaluated against a reference standard that was influenced by the result of this item (incorporation bias), and additional assays that factored into the reference standard were performed depending on the responses to these items (verification bias) (20).

The 2004 study additionally reports a sensitivity of 90.8% and a specificity of 86.6% for the PFA collagen–epinephrine test (Fig. 3 of the 2004 publication). While the 2004 publication itself does not describe in detail the reference used for this analysis, the manufacturer’s instructions for use, describe it as “impaired platelet function based on all laboratory data (except PFA-100 System results), and patient medication history” (14). However, the tests used as reference (i.e., assays other than PFA) were in part performed after, and with knowledge of, the PFA result. The authors also mention that in 14 patients antiplatelet drug use was “suspected after testing and confirmed upon requestioning”. A patient with an abnormal PFA result therefore had a considerably greater chance of being investigated further and confirmed as having “impaired haemostasis” than a patient with a normal result. In the terminology of QUADAS-2 (20), this constitutes verification bias. In addition, the cut-off underlying these figures is not stated and was selected by ROC analysis within the same dataset, i.e., optimized *post hoc*, which often inflates performance metrics (20).

To illustrate that the detection rates reported in the 2004 study can be generated by a self-referential approach without a diagnostic relationship, we conducted the following experiment: A dataset comprising 256 “patients” and seven “assays” was generated in Microsoft Excel, with each cell populated by random numbers uniformly distributed between 0 and 1 (the corresponding file is available as Supplementary Material). Cut-offs were then defined (using an artificial intelligence tool, GPT-5.3) such that each assay yielded the same proportion of positive results as reported in the 2004 study. In addition, the dataset was minimally adjusted to ensure that each patient classified as having “impaired hemostasis” exhibited at least one abnormal test result, consistent with the original study definition. Based on this synthetic dataset, the “detection” of “impaired hemostasis” was calculated. Not surprisingly, the synthetic dataset “detected” “impaired hemostasis” equally well as reported in the 2004 study (Table 1). This simple experiment shows that, given a suitable cut-off and a self-referential case definition, random numbers can produce any desired “sensitivity” — a statistical perpetuum mobile (21). This analysis demonstrates the statistical problem of self-referential approaches; it is not a statement about the raw data of the 2004 study or about PFA as a method.

**Table 1:** Tests, with cut-offs calculated by artificial intelligence based on random numbers (between 0−1), to achieve the same proportion of positives as in (1). BT: bleeding time, PC: platelet count.

test	cut-off	positive n	detection of “impaired hemostasis”
PFA C-epinephrine	0.01176	250	97.7%
PFA C-ADP	0.21360	199	77.7%
BT	0.25902	188	73.4%
vWF:Ag	0.86891	39	15.2%
aPTT	0.91758	24	9.4%
PT	0.95756	10	3.9%
PC	0.99065	2	0.8%

## Discussion

The aim of this Perspective is not to assign blame to the authors of the 2004 publication (1). We recognize the value of the article with regard to the questionnaire presented and the observational data reported. Scientific manuscripts are not infallible, and critical appraisal is an integral part of the scientific process. The authors should be credited for transparently outlining the basis and reasoning underlying their conclusions.

### What are the consequences of our observations?

If the 2004 article (1) had been largely forgotten, the issues discussed here would be of limited relevance. However, as its findings remain incorporated into the instructions for use of two widely applied assays and continue to be cited more than two decades after publication, we believe that this topic warrants re-examination and, where appropriate, corrective action. In this sense, the publication has, to some extent, become a victim of its own success.

The article contains 3 main elements: a) a standardized questionnaire, b) observational data in a large patient cohort, and c) sensitivity and specificity calculations. According to our assessment only the latter is invalid, while the other two sections may remain relevant. Also, our evaluation concerns the diagnostic accuracy estimates of the 2004 study only. It does not imply that the PFA assays lack diagnostic value, and it does not affect the findings of other published studies on these assays, whose methodological rigor we did not evaluate.

### Reference in the instructions for use for the PFA collagen-epinephrine and collagen-ADP cartridges

The instructions for use (13) state: “The use of a standardized questionnaire in combination with the COLL EPI CARTRIDGE provided a high detection rate of impaired hemostasis in a pre-surgical screening approach including more than 5 000 patients scheduled for elective surgery. The vast majority of hemostasis defects identified affected the primary hemostasis; 98% of all hemostasis defects were identified by the COLL EPI CARTRIDGE” [reference to (1)].

Both the reported “high detection rate of impaired hemostasis” and the identification of “98%” of defects are derived from the 2004 study. We therefore recommend that the manufacturer of the respective analyzer reassess the use of this evidence.

The 2004 study is also cited in the edition of the instructions for use for the PFA-100 reagents that the manufacturer provides for the US market (14).

### References to the 2004 article in selected guidelines and review articles

A position paper by the Romanian “Patient Blood Management Initiative Group” (9) cites the 2004 study in the context of recommending specialist consultation when abnormalities in hemostasis are suspected. This use does not rely on the problematic aspects of the article. Similarly, a more recent review by Erdoes et al. (4) references the study in support of preoperative screening for specific coagulopathies, which remains a valid clinical recommendation. In a position paper by an interdisciplinary expert group on patient blood management in India (22), the study is cited in relation to the recommendation to obtain a patient's bleeding and medication history prior to surgery.

In contrast, a recent review on the PFA method (23) cites the 2004 study to support the statement that “as a replacement for the ’skin bleeding time’, normal closure times can be used as a marker of low surgical bleeding risk.” This claim appears to rely on the biased estimates of the 2004 study. In an earlier review, the same author (5) noted: “Impressively, Koscielny et al. reported large studies and concluded that use of standardized questionnaires and, if indicated, C/Epi CT and/or other specific tests (e.g., VWF testing), ensured detection of impaired hemostasis in advance of surgical procedures… However, other studies assessing bleeding risk in various settings have provided less convincing and contradictory results.” While the author acknowledged inconsistencies in reported diagnostic performance, the statistical basis of the 2004 study was not critically examined.

Similarly, a review on total hip arthroplasty in patients with coagulopathy (24) states: “Bearing in mind the study of Koscielny et al., all patients with a positive history of hemorrhage can also be identified with the PFA-100 test… A standardized hemorrhage history in accordance with Koscielny et al.'s questionnaire together with the PFA-100 test can reliably detect most cellular disorders in hemostasis.” These statements again appear to rely on the same methodological limitations of the 2004 study.

Taken together, these examples illustrate that the 2004 study continues to influence the field of primary hemostasis diagnostics.

## Conclusion

The principal implication of our findings is that the widely cited 2004 estimates cannot be used as evidence of diagnostic accuracy, because the target condition was defined using the tests whose performance was subsequently evaluated.

### Further learnings

This case underscores the importance of continuously scrutinizing the scientific validity of published evidence, including studies that have become widely cited over time. When developing review articles and clinical guidelines, key references should be critically appraised with respect to their methodological robustness.

The impact of the 2004 publication can, in part, be attributed to its alignment with the needs of the time: it introduced a structured questionnaire, reinforced the importance of clinical history, and appeared to offer a practical solution for the assessment of primary hemostasis. In this sense, it aligned closely with the zeitgeist and aspirations of the scientific and clinical communities.

Findings that seem to offer solutions to pressing scientific or clinical problems may attract less critical scrutiny from authors, reviewers, and readers (25).

## Data Availability

The synthetic dataset generated for this study is provided as Supplementary Material. All other data discussed are derived from previously published sources, which are cited in the article.
